
JOURNAL INFORMATION
==============================
NLM Title Abbreviation: Behav Neurol
Journal ID: Behav Neurol
NLM Journal ID: 8914585
Journal ID: BN

Behavioural Neurology

ISSN: 0953-4180
EISSN: 1875-8584
Publisher: Wiley

ARTICLE INFORMATION
==============================
PMCID: PMC5434403
PMID: 21422549
DOI: 10.3233/BEN-2010-0289
Article ID: 829571
Article version: 1
Subjects: Research Article

Adaptive Testing Combines Precision with Brevity in the Grading of Cognitive Impairment

Wouters Hans 1
Appels Bregje 4
Van Campen Jos 5
Lindeboom Robert 1
Buiter Maarten 1
Zwinderman Aeilko H. 1
van Gool Willem A. 2
Schmand Ben 2 3
1 Department of Clinical Epidemiology Biostatistics and Bioinformatics Academic Medical Center Amsterdam The Netherlands
2 Department of Neurology Academic Medical Center Amsterdam The Netherlands
3 Department of Psychology University of Amsterdam Amsterdam The Netherlands
4 Department of Medical Psychology Slotervaart Hospital Amsterdam The Netherlands
5 Department of Geriatric Medicine Slotervaart Hospital Amsterdam The Netherlands
Print publication date: 2010
Electronic publication date: 2011 Mar 7
Volume: 23
Issue: 4
First page: 181
Last page: 183
Received 2011 Jan 31; Accepted 2011 Jan 31
Copyright: Copyright © 2010 Hindawi Publishing Corporation and the authors.
Copyright year: 2010
License: This is an open access article distributed under the Creative Commons Attribution License, which permits unrestricted use, distribution, and reproduction in any medium, provided the original work is properly cited.
License URL: https://creativecommons.org/licenses/by/3.0/

==============================
Behavioural Neurology 23 (2010) 181–183
DOI 10.3233/BEN-2010-0289
IOS Press

### Adaptive testing combines precision with brevity in the grading of cognitive impairment

Hans Wouters[a], Bregje Appels[d], Jos Van Campen[e], Robert Lindeboom[a], Maarten Buiter[a], Aeilko H. Zwinderman[a], Willem A. van Gool[b] and Ben Schmand[b,c]
[a]*Department of Clinical Epidemiology, Biostatistics and Bioinformatics, Academic Medical Center, Amsterdam, The Netherlands*
[b]*Department of Neurology, Academic Medical Center, Amsterdam, The Netherlands*
[c]*Department of Psychology, University of Amsterdam, Amsterdam, The Netherlands*
[d]*Department of Medical Psychology, Slotervaart Hospital, Amsterdam, The Netherlands*
[e]*Department of Geriatric Medicine, Slotervaart Hospital, Amsterdam, The Netherlands*

### 1. Objective

In elderly patients, dementia severity and level of cognitive impairment are typically graded with total scores on fixed tests. However, this approach is inflexible. Every test item has to be administered to all individuals in order to obtain comparable total scores. As a result, longer tests that are to be preferred because of their precision are because of their duration abandoned by many clinicians. Instead, many clinicians choose brief instruments which have however less precision especially in the grading of early dementia.

Computerized Adaptive Testing (CAT) is a potential solution to this problem. It combines the precision of longer tests with the brevity of shorter ones. Rather than administering every item to every individual to estimate his or her cognitive ability level, CAT tailors the test by selecting a more difficult item after a correct response and an easier one after an incorrect response. As a result, CAT e.g. skips the item "what date is it today?" when the patient does not know the answer to the item "what day it is today?" In this study we examined the adaptive administration of (i) a set of 47 CAMCOG items (C-47) and (ii) a set of 51 items selected from the CAMCOG, the ADAS-cog and a neuropsychological test battery (C-Plus).

### 2. Methods

### 2.1. Participants

We studied patients from a geriatric day clinic and their partners. Participants with impaired vision or hearing, illiteracy, functional problems of the dominant hand or who could not be tested for other reasons were excluded. Partners were also excluded if they were younger than 55 and if they had relevant memory complaints. A total of 84 participants were included of whom 67 were patients (demented $n = 22$, MCI $n = 21$ and non-demented $n = 24$) and 17 were partners. Written informed consent was obtained for all participants. The local medical ethical committee approved the study.

### 2.2. Adaptive cognitive testing

Participants were randomly assigned to the C-47 ($n = 45$) or the C-Plus ($n = 39$). A CAT algorithm was used to tailor the C-47 or C-Plus to each individual participant. Using standard sets of six to eight items, cognitive ability was provisionally estimated. Subsequently, the CAT algorithm estimated a patient's ability level along with a standard error as a measure of reliability. After each response, the CAT algorithm updated the ability estimate and the standard error by selecting a more difficult item in case of a correct response or an

ISSN 0953-4180/10/$27.50 © 2010 – IOS Press and the authors. All rights reserved

Table 1
Test length of CAT compared to whole tests and validity coefficients between CAT and whole tests

| | CAT vs. Whole C-47 | CAT vs. Whole C-Plus |
|---|---|---|
| Median number of items (interquartile range) | 24 (20–25) vs. 47 | 24 (18–25) vs. 51 |
| % reduction in number of items | 49 | 53 |
| Median minutes of testing time (interquartile range) | 20 (17–23) vs. 35 (30–40) | 48 (43–53) vs. 104 (92–111) |
| % reduction in testing time | 43 | 54 |
| Agreement between CAT and Whole Test estimates of ability (Intraclass Correlation) | 0.99 | 0.98 |
| Rank correlation of CAT ability estimate with: | | |
| MMSE | 0.89 | 0.80 |
| Original CAMCOG | 0.93 | −* |
| IQCODE | −0.76 | −0.54 |

Abbreviations: CAT, Computerized Adaptive Testing; C-47 & C-Plus, see text; MMSE, Mini Mental State Examination; CAMCOG, Cambridge Cognitive Examination; IQCODE, Informant Questionnaire on Cognitive Decline in the Elderly. * original CAMCOG not administered in C-Plus condition.

easier one in case of an incorrect response. The item difficulty levels used by CAT were previously estimated and examined for their validity with Rasch analysis using existing data [1] (for C-47, see [2], for C-Plus, results available on request). The CAT algorithm terminated after reaching a maximum of 25 items or a standard error reflecting a reliability of 0.90. The remaining items not selected by CAT were subsequently administered to obtain conventional cognitive ability estimates based on the whole item sets of the C-47 or C-Plus.

### 2.3. Statistical analyses

The median number of items and testing time needed by CAT were calculated and compared to those of the whole C-47 or C-Plus. Intraclass correlations were calculated to examine the agreement between estimates as obtained with CAT and with either the whole C-47 or the C-Plus. The concurrent validity of the CAT estimates with the MMSE [3], the original CAMCOG [4] and IQCODE [5] was examined with Spearman's rank correlations.

### 3. Results

### 3.1. Sample characteristics

Patients with MCI or dementia had lower MMSE scores than non-demented participants (Median scores 27 and 20, interquartile ranges 14–30 and 12–24 versus Median score 29, interquartile range 24–30). They did not differ substantially from non-demented participants with respect to age, level of education or depressive symptoms. However, patients with MCI were less likely to be female and less likely to have a history or presence of stroke, TIA or TBI (data available on request).

### 3.2. Test reduction & agreement between adaptive testing and whole test

With CAT, a substantial reduction in number of items and testing time was obtained compared to the whole C-47 and C-Plus (see Table). The percentage reduction of testing time accomplished with CAT was 43% compared with the whole C-47 and 54% compared with the whole C-Plus. This was accompanied by excellent agreement between the abilities as estimated by CAT and as estimated by the complete C-47 and C-Plus item sets (see Table). Exploratory analyses (results available on request) showed that these results were sustained for CAT reduced test lengths up to 15 items.

### 4. Conclusions

Our findings suggest that long and precise cognitive tests can be administered much more efficiently by selecting only items of appropriate difficulty for individual patients using Computerized Adaptive Testing

(CAT). Substantial reductions in the number of items and testing time needed by CAT were found compared to the whole tests (C-47 and C-Plus). Even so, the CAT estimated abilities were in excellent agreement with those based on all items of either the whole C-47 or C-Plus. Generally, concurrent validity with the MMSE, CAMCOG and IQCODE was high. These findings confirm our previous findings based on retrospective data obtained from AD patients and stroke or VaD patients [6,7].

The results are clinically relevant. CAT combines brevity with precision in the grading of dementia severity and cognitive impairment. In busy clinical settings or large scale epidemiological studies, CAT facilitates as such the use of precise instruments that in their entire form would be too long to administer.

This study had some limitations. The sample size was moderate. Our findings pertain to the grading of global cognitive impairment. Therefore, adaptive testing cannot replace a full domain-specific neuropsychological examination. Further studies should be conducted to examine if adaptive cognitive testing improves the detection of treatment effects in clinical research compared to standard instruments, possibly by reducing fatigue and test burden in impaired patients and by including items sensitive at the extremes of the scale while keeping testing efficiently. Taken together, our findings show that adaptive testing combines precision with brevity in the grading of (early) cognitive impairment.

### References

[1] N.D. Verhelst and C.A.W. Glas, The one parameter logistic model, in: *Rasch Models, Foundations, Recent Developments and Applications*, G.H. Fischer and I.W. Molenaar, eds, Springer Verlag, New York, 1995, pp. 215–237.

[2] R. Lindeboom, B. Schmand, R. Holman, R.J. de Haan and M. Vermeulen, Improved brief assessment of cognition in aging and dementia, *Neurology* **63** (2004), 543–546.

[3] M.F. Folstein, S.E. Folstein and P.R. McHugh, "Mini-mental state", A practical method for grading the cognitive state of patients for the clinician, *Journal of Psychiatric Research* **12** (1975), 189–198.

[4] M. Roth, E. Tym, C.Q. Mountjoy, F.A. Huppert, H. Hendrie, S. Verma et al., CAMDEX. A standardised instrument for the diagnosis of mental disorder in the elderly with special reference to the early detection of dementia, *British Journal of Psychiatry* **149** (1986), 698–709.

[5] A.F. Jorm, R. Scott, J.S. Cullen and A.J. MacKinnon, Performance of the Informant Questionnaire on Cognitive Decline in the Elderly (IQCODE) as a screening test for dementia, *Psychological Medicine* **21** (1991), 785–790.

[6] H. Wouters, A.H. Zwinderman, W.A. Van Gool, B. Schmand and R. Lindeboom, Adaptive cognitive testing in dementia, *International Journal of Methods in Psychiatric Research* **18** (2009), 118–127.

[7] H. Wouters, I. de Koning, A.H. Zwinderman, W.A. Van Gool, B. Schmand, M. Buiter et al., Adaptive Cognitive Testing in Cerebrovascular Disease and Vascular Dementia, *Dementia and Geriatric Cognitive Disorders* **28** (2009), 486–492.
